# Opioid-Induced Constipation in Advanced Cancer Patients

**DOI:** 10.7759/cureus.14386

**Published:** 2021-04-09

**Authors:** Nabil ALMouaalamy

**Affiliations:** 1 Oncology Department/Palliative Care, Princess Noorah Oncology Center, King Abdulaziz Medical City, Ministry of National Guard-Health Affairs, Jeddah, SAU; 2 Research, King Abdullah International Medical Research Center, Jeddah, SAU; 3 College of Medicine, King Saud Bin Abdulaziz University for Health Sciences, Jeddah, SAU

**Keywords:** opioids, cancer, constipation

## Abstract

The present study discusses opioid-induced constipation (OIC) in advanced cancer patients, focusing on the OIC definition, pathophysiology, and treatment. OIC is any change from baseline defecation patterns and bowel habits that developed after starting opioid therapy. The condition is characterized by bowel frequency reduction, worsening or development of straining, a sensation of incomplete defecation, or distress associated with bowel habits. OIC is common in advanced cancer patients, with a prevalence of approximately 51%-87% in patients taking opioids for pain management. Patients are likely to experience severe distress, work productivity reduction, poor quality of life, and increased healthcare utilization. OIC has a complex pathophysiology that involves propulsive and peristalsis impairment, intestinal mucosal secretion inhibition, intestinal fluid absorption enhancement, and anal sphincters function impairment. The Rome III criteria are used to assess and diagnose clinical OIC and can also be diagnosed through the Patient Assessment of Constipation (PAC) measures, including the symptom survey (PAC-SYM) and quality of life survey (PAC-QOL). Non-pharmacological treatment of OIC involves lifestyle habits and dietary adjustments, although these interventions might be insufficient to manage the condition. Pharmacological treatments involve the use of traditional laxatives and newer agents like peripherally acting mu-opioid receptor agonists (PAMORAs), including naldemedine, naloxegol, and methylnaltrexone. More novel treatments for OIC that target the pathophysiology are still needed and should be studied carefully for safety and efficacy.

## Introduction and background

Pain is a serious concern for people who are diagnosed with different forms of cancer. Chiefly, pain prevalence rates are 39.3% following curative therapy, 66.4%-80% in advanced cancer, and 55% during anticancer therapy [1]. There has been increased attention on pain in cancer patients because it affects the quality of life and is associated with many psychosocial responses. As pain is highly prevalent among cancer patients, pain alleviation is a critical treatment goal. The American Society of Clinical Oncology recommends the use of opioids to manage pain in selected cancer patients who are unresponsive to conservative pain management approaches and continue to experience functional impairment or distress [2]. Opioids are recommended for various types of cancer pains, such as neuropathic, visceral, and somatic pain, because of ease of titration, efficacy, and favorable safety profile [3].

Even though opioids are effective, they are linked to various adverse events, such as opioid-induced constipation (OIC), abdominal discomfort, oesophageal reflux, dry mouth, nausea, and vomiting [4]. Most adverse events, such as vomiting and nausea, disappear after few days, but OIC can persist throughout the opioid treatment period. Opioids prescription should incorporate necessary precautions to prevent adverse events, abuse, and addiction. OIC has a substantial effect on the quality of life for cancer patients on opioid treatment. Cancer patients tend to associate constipation with severe distress. Moreover, patients are likely to report work productivity reduction, low quality of life, and healthcare utilization increase. Cancer patients tend to discontinue or avoid opioid therapy because of OIC, which might cause them to sacrifice effective pain control to prevent constipation [3]. Therefore, OIC is emerging as a key aspect in cancer patients who use opioids for pain management. This paper discusses OIC, its pathology, and treatment options.

## Review

Definitions

OIC is the most prevalent form of opioid-induced bowel disorder (OIBD). Opioid action on the gastrointestinal (GI) tract and central nervous system (CNS) or the unintended consequences of opioid therapy on the GI tract are collectively referred to as OIBD [5]. OIBD develops when opioids disrupt normal GI function by binding to opioid receptors [6]. OIC is defined by Camilleri M et al. as any change from baseline defecation patterns and bowel behaviors that developed after starting opioid therapy. This change is characterized by one of the following: 1) bowel frequency reduction, 2) worsening or development of straining, 3) a sensation of incomplete defecation, and 4) harder stool consistency [7]. Any of these changes can be a symptom of OIC if they develop upon opioid therapy initiation. A patient might also experience fecal impaction characterized by overflow incontinence, whereas other patients can experience symptoms consistent with overlapping OIBD such as bloating, nausea, and reflux [5].

OIC can be frequent in advanced cancer patients using opioid therapy to manage pain. There is no consensus on the actual OIC frequency among advanced cancer patients. Lacy et al. indicate that OIC prevalence is about 41% in those with chronic noncancer pain placed under opioid therapy [5]. Among cancer patients using opioids for pain control, the prevalence of constipation is almost 94% [5]. In another study, Farmer et al. observe that OIC happens in 51%-87% of cancer patients under opioid therapy and 41%-57% patients taking the therapy for chronic noncancer pain [8]. Even though OIC is a prevalent reason for constipation, other factors might influence constipation occurrence or worsen OIC symptoms in cancer patients. The differential diagnosis of OIC is important to determine the specific cause of constipation and offer effective treatment. Another important definition is laxative-refractory OIC, which is defined as inadequate laxative response with severe symptoms of constipation (bowel function index (BFI) ≥ 30), despite the scheduled use of two laxatives from two or more laxative classes for a minimum of four days within a two-week period [9].

Pathophysiology

OIC develops due to propulsive and peristalsis impairment, intestinal mucosal secretion inhibition, intestinal fluid absorption enhancement, and anal sphincters impairment. The disturbances in normal function or impairment occur because of opioid receptors' activation within the GI by exogenous opioids [6].

Opioid-induced gut dysmotility

OIC results from opioid-mediated activity on receptors in the GI and even in the CNS [3]. The primary opioid receptors are μ‐, κ‐, and δ‐, and these receptors are dispersed across the GI tract [8]. The distribution of opioid receptors varies significantly based on the layer and specific region of the gut. High levels of μ‐ and κ‐ are found within the proximal colon and stomach [8]. On the other hand, μ‐receptors are mainly dispersed throughout the GI tract and largely localized with submucosal neurons and myenteric and on immune cells within the lamina propria [6].

Opioid receptors and opioid peptides are expressed by different intestinal muscle cells and enteric neurons. Upon release from neurons, opioid peptides serve as transmitters in the enteric modulation of peristalsis and propulsive motility. Chiefly, the impact of this mechanism is twofold. Firstly, it attenuates the normal peristalsis and propulsion. Secondly, it triggers non-propulsive motility patterns and tonic spasms in the colon and small intestines [3]. The cascade impact is a gastric-emptying delay, abdominal cramping, deceleration of intestinal transit, and eventually constipation.

Opioids impact on the mucosal secretion

About nine to 10 liters of fluid are secreted from the GI tract daily [8]. Opioids have a major impact on the absorptive and secretory GI's function via several mechanisms. Opioid receptor activation within the GI tract inhibits electrolyte and submucosal water secretion [3]. Also, opioid receptor activation raises fluid absorption from lumina space and retention. Treatment with opioids increases sympathetic tone, which decreases secretions into the lumen [3]. Ultimately, these effects increase the absorption of fluid from the lumen, and stools become hard and dry.

Opioids impact on GI sphincters

Opioids have a profound impact on defecation reflexes [3]. A human's GI tract has about six functionally or anatomically characterized sphincters, including the lower and upper esophageal sphincters, sphincter of Oddi, pylorus, anal sphincters, and ileocaecal valve [8]. Opioids can modulate the functioning of all these sphincters, which impact the GI tract's integrity. The anal sphincters play an important role in the OIC's pathology. Opioid-induced impairment of anorectal function is marked by an increase in internal anal sphincter contraction. The increased internal anal sphincter contraction leads to straining, hemorrhoids, and a feeling of incomplete defecation [8]. In particular, anal sphincter impairment decreases anal and rectal sensitivity to the rectal vault and creates a sense of anal blockage, common among patients with OIC [3]. A sense of incomplete defecation, straining, and hemorrhoids together can result in severe challenges with defecation, and colonic perforation might happen in the worst scenario. For example, loperamide tends to increase the internal sphincter tone, and about a third of patients placed under opioids treatment report a feeling of anal blockage even after laxative treatment [8]. These findings have been reproduced in recent studies. Poulsen et al. found that oxycodone impairs anal sphincter relaxation, and slow-release naloxone can reverse this effect [10].

Assessment and diagnosis of OIC

Clinical OIC Assessment and Diagnosis

Patients who are being treated with opioids might present with constipation because of several factors that might not be identified on initial assessment [8]. Opioid treatment might not be the primary cause but may worsen the symptoms. Clinicians should take an in-depth patient history when assessing patients who present with constipation. A clinician should focus on the normal bowel habit and changes after opioid therapy introduction. Patients are likely to identify potential changes in bowel movement that occurred when opioid treatment was initiated. Comprehensive drug history is required during the assessment to determine drugs that may be causing constipation.

The patient's health history and current medications might be insufficient to assess and diagnose OIC in cancer patients. Rome III criteria (Table 1) can be used for OIC diagnosis in clinical settings [5]. The Rome III criteria have proven effective in defining OIC in clinical practice and clinical trials [11]. Even though Rome III criteria have been created for functional constipation, they offer a standardized OIC definition. This assessment standard comprises straining at defecation, hard stools, anorectal obstruction or a feeling of incomplete defecation, reliance on manual aids to facilitate stools passage, and passing under three stools a week [11].

**Table 1:** Rome III diagnostic criteria for opioid-induced constipation Source [5]

Diagnostic Criteria for Opioid-Induced Constipation
1. New, or worsening, symptoms of constipation when initiating, changing, or increasing opioid therapy that must include 2 or more of the following:
a. Straining during more than one-fourth (25%) of defecations
b. Lumpy or hard stools (BSFS 1-2) more than one-fourth (25%) of defecations
c. Sensation of incomplete evacuation more than one-fourth (25%) of defecations
d. Sensation of anorectal obstruction/blockage more than one-fourth (25%) of defecations
e. Manual maneuvers to facilitate more than one- fourth (25%) of defecations (eg, digital evacuation, support of the pelvic floor)
f. Fewer than three spontaneous bowel movements per week
2. Loose stools are rarely present without the use of laxatives

Furthermore, digital rectal examinations are recommended in patients consulting for opioid-induced constipation. Digital rectal examinations are important for clinicians to exclude anorectal malignancy, minor anal pathologies, such as anal fissure and fecal impaction, which likely worsen symptoms [8]. Systematic bowel function review is recommended for patients starting opioids treatment and maintaining opioids treatment due to OIC prevalence.

Patient-Reported OIC Outcome Measures

Several scales are available to help in the diagnosis of OIC among cancer patients. Patient-reported scales for assessing OIC have been validated in patients with different cancers. The Bowel Function Index (BFI) has received a lot of attention because of its simplicity [3]. Importantly, the BFI is a survey administered by a clinician that consists of three questions to assess defecation ease, the sense of incomplete defecation, and the patient's personal judgment concerning constipation [3]. A patient scores the questions based on his or her personal experiences in the last seven days. The three questions are scored through a numeric scale ranging from 0-100, and the mean score is treated as the final score. Zero (0) means no symptom while 100 means severe symptoms. The BFI is reliable and seems responsive to changes in OIC severity [3]. A 12-point change in the BFI scale is considered a clinically important constipation change. A consensus panel on OIC and other opioid-associated negative events recommends treatment for patients with a BFI score of ≥ 30 [12]. The consensus panel advocates the BFI scale application because of its ease of use (See Table 2).

**Table 2:** Bowel function index

Item	Question	Scale
1	During the last 7 days, how would you rate your ease of defecation on a scale from 0 to 100?	0 = easy or no difficulty 100 = severe difficulty
2	During the last 7 days, how would you rate your feeling of incomplete bowel evacuation on a scale from 0 to 100?	0 = not at all 100 = very strong
3	During the last 7 days, how would you rate your constipation on a scale from 0 to 100?	0 = not at all 100 = very strong
Total score		Mean of 3 scores

The Patient Assessment of Constipation (PAC) is widely used to assess OIC in cancer patients. Among the common PAC, measures include the "symptom survey (PAC-SYM) and quality of life survey (PAC-QOL)" [3]. The PAC-SYM questionnaire was created via psychometric assessment of patients with chronic constipation. This questionnaire is considered effective in evaluating the severity of symptoms in patients with OIC. The survey has 12 items divided into 3 symptom subscales, including rectal (3 items), stool (5 items), and abdominal (4 items) [13]. Items of this scale are scored through five-point Likert scales, ranging from 0-4. In this case, 0 shows the absence of symptoms, while 4 indicates very severe symptoms. The total score is divided by a number of questions to get an average total score, ranging from 0-4 [12]. A low average score shows a low symptom burden.

On the contrary, PAC-QOL consists of 28 items that are divided into psychological discomfort, physical discomfort, dissatisfaction, and concerns and worries [3]. These four domains are meant to assess the extent to which OIC symptoms are impacting a patient. Rumman et al. indicate that PAC is beneficial because it is comprehensive and assesses a patient's quality of life and OIC symptoms burden [3]. The patient-reported outcome measures have become important because of the increasing concern of OIC among cancer patients. Nevertheless, most patient-reported outcome measures are too complex for routine clinical practice.

Treatment

Non-Pharmacological Treatment

Dietary interventions might improve OIC symptoms and improve a patient's quality of life. According to Gregorian et al., adjustments in lifestyle habits and diet can substantially improve OIC and other forms of constipation [14]. A patient can improve constipation by consuming 25-30 g of soluble fiber daily, having a balanced diet, sufficient fluids, regular aerobic exercise, regular meal patterns, and avoiding fat, heavy meat, flatulent foods, and insoluble fiber [5]. Rumman et al. acknowledge that non-pharmacological treatments alone might be insufficient to OIC management but act as pharmacology treatment adjuncts [3]. Patient education concerning activity and fluid intake should be emphasized where possible to help patients prevent OIC.

Pharmacological Treatment

The American Gastroenterological Association (AGA) recommended the use of laxatives as the first-line in patients with OIC [9]. Several studies show that stimulant laxatives (senna, bisacodyl, and picosulphate) and osmotic laxatives (polyethylene glycol) should be the first choice in OIC patients [15-16]. These medications can be used as first-line options for OIC management (see Table 3). However, laxatives have several adverse side effects such as gas, fullness/bloating, and defecation urgency [8].

**Table 3:** Different classes of agents for opioid-induced constipation and mechanisms of action Note. Adapted from [9] AGA: American Gastroenterological Association; PEG: polyethylene glycol; PAMORA: peripherally acting mu-opioid receptor agonist

Class/type	Examples	Mechanism of action	Role in cancer patients
Traditional laxatives			
Bulking Agents	Soluble (e.g., psyllium, pectin) Insoluble (methylcellulose)	Induce a stretch reflex in the intestinal wall, which increases the colonic motility, water absorption, and bacterial proliferation in the colon, leading to softening the stool and a smoother process.	Bulk laxatives are not effective for already-constipated cancer patients.
Osmotic	PEG, lactulose, magnesium citrate, magnesium hydroxide	Draw water into intestine to hydrate and soften stool	Yes, PEG is one of the first-line recommendations
Stimulant	Bisacodyl, sodium picosulfate, senna	Irritate sensory nerve endings to stimulate colonic motility and reduce colonic water absorption	Yes, first-line recommendation
Detergent/surfactant stool softeners	Docusate	Allow water and lipids to penetrate the stool to hydrate and soften fecal material	Patients must have enough fluid intake with these agents and it doesn’t work alone, Stimulant have to be added.
Lubricant	Mineral oil	Lubricate the lining of the gut to facilitate defecation	
PAMORAs	Naldemedine	Block μ-opioid receptors in the gut, thereby effectively restoring the function of the enteric nervous system	Yes
	Naloxegol		Yes
	Methylnaltrexone		Yes
Intestinal secretagogues	Lubiprostone	Act on chloride channels or guanylate cyclase receptors in enterocytes to stimulate fluid secretion into the intestinal lumen	No, AGA. No recommendation due to evidence gap.
Selective 5-HT agonists	Prucalopride	Activate 5-HT4 receptor, leading to increased colonic motility and accelerated transit	No, AGA. No recommendation, due to evidence gap.

**Figure 1:**
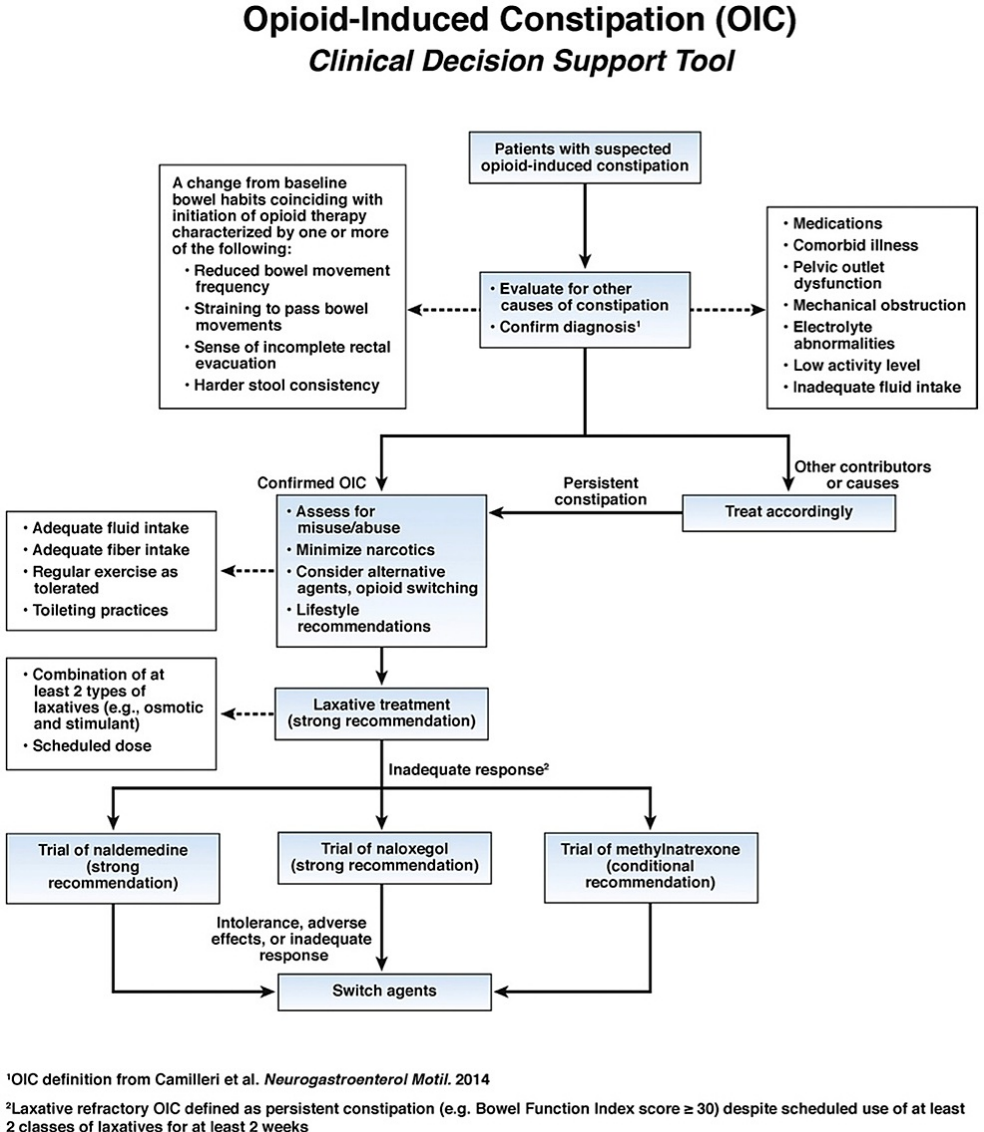
AGA OIC clinical decision tool Source [9] AGA: American Gastroenterological Association; OIC: opioid-induced constipation

Also, opioid switching or using another opioid that is less constipating is a solution [17]. For example, transdermal opioids like fentanyl may cause less constipation than morphine in oral or parenteral preparations [18]. Also, the use of combination opioid agonist/antagonist agents (eg, oxycodone + naloxone) may decrease the risk of constipation [19]. The Food and Drug Administration (FDA)-approved prescription products for OIC are lubiprostone, naloxegol, methylnaltrexone, and naldemedine [14]. It is recommended to use the AGA OIC clinical decision tool when trying to approach a patient with OIC (Figure 1) [9].

Lubiprostone

Lubiprostone is a Cl‑ channel activator that increases the secretion of intestinal fluid and enhances fecal transit within the GI tract [20]. This product also alters gut mucus composition and flora to produce an anti-inflammatory luminal milieu [3]. The recommended dosage of this product is 24 mcg twice each day and should be taken with water and food [14]. Patients with irritable bowel syndrome can take 8 mcg capsules of lubiprostone. Due to dosage variations, it is important for pharmacists to ensure that they prescribe the correct dose. Lubiprostone has been found to improve bowel movement and stool consistency significantly as compared to placebo, although it does not relieve abdominal pain [21].

Among the most prevalent side effects of using lubiprostone for OIC treatment are diarrhea and nausea [20]. Furthermore, hypotension, dyspnea, and syncope are likely to occur after the completion of the first dose. Most of these effects disappear on their own but might reoccur in subsequent doses. Dose modification is not needed for patients with renal impairment, but adjustments may be essential for patients with hepatic impairment. The likelihood of drug-drug interactions is minimal, as CYP450 isoenzymes do not modulate lubiprostone metabolism [14]. Lubiprostone can be tolerated for about 13 months, meaning it is effective for long-term use.

Peripherally Acting Mu-Opioid Receptor Agonists (PAMORAs)

Methylnaltrexone, naldemedine, and naloxegol are a class of PAMORAs since they block peripheral mu-opioid receptors and do not inhibit opiates' analgesic effects on the mu receptor in the CNS [14]. Nevertheless, patients taking PAMORAs have a risk of opioids withdrawal and need to be monitored for anxiety, rhinorrhea, hyperhidrosis, and chills. More importantly, opioid antagonists like naltrexone and naloxone should not be used together with PAMORAs due to the risk of addiction and withdrawal [14]. A patient should only be prescribed PAMORAs if they have taken opioids for four weeks and laxatives need to be discontinued [14]. Each PAMORA has distinct attributes despite being in the same class.

Methylnaltrexone

Methylnaltrexone is available in subcutaneous (SC) injection and oral formulations. The N-methyl group in this drug inhibits the capacity to cross the blood-brain barrier because of its low lipid solubility and polarity [8]. SC-injection formulation was approved for OIC treatment in advanced cancer patients who failed to respond sufficiently to laxatives. The recommended doses of SC injection are 12 mg for 114 kg and 8 mg for 62 kg [8].

The recommended dose is 450 mg once per day for oral methylnaltrexone and should be taken 30 min before the first meal [14]. Siemens and Becker designed a meta-analysis study to identify the safety and effectiveness of methylnaltrexone in OIC patients [22]. Based on the findings, patients being treated with methylnaltrexone had rescue-free bowel movement only four hours after the initial dose, higher stool regularity, and required less time to laxation than placebo [22]. These patients also had greater levels of satisfaction, better outcomes, and minimal adverse events.

Naloxegol

Naloxegol is approved for OIC treatment in patients with chronic pain related to cancer and non-cancer pain. Although the recommended dose is 25 mg per day, it can be tapered to 12.5 mg if not well-tolerated [14]. Oral intake is recommended, but this product might be crushed and administered through a nasogastric tube where necessary. Just like methylnaltrexone, naloxegol should be taken on an empty stomach. A patient can take this medication one hour before eating or two hours after eating [14]. Patients taking naloxegol should avoid certain products such as grapefruits and juice. The side effects of taking naloxegol are flatulence, nausea, vomiting, abdominal pain, headache, diarrhea, and arthralgia [14]. Double-blind studies meant to study the safety and efficacy of naloxegol have found that response rates are higher in patients treated with naloxegol than placebo without decreasing opioid-mediated analgesia [23]. They have concluded that the treatment is effective for OIC treatment. A newly published observational study for using naloxegol in 126 cancer patients found that it did improve quality of life and relieve constipation, which lasted for the whole follow-up period, which was one year [24].

Naldemedine

As naldemedine is structurally comparable to naltrexone, it has been categorized as a Schedule II controlled product. A patient can take a dose of 0.2 mg per day with or without an empty stomach [14]. Even though renal adjustment is unnecessary, patients with hepatic impairment should avoid this product. Patients are likely to experience a range of side effects, including gastroenteritis, abdominal pain, nausea, and diarrhea. P-glycoprotein inhibitors and strong and moderate CYP3A4 inhibitors might raise naldemedine concentrations [14]. Patients should be monitored for adverse reactions if they are taking these medications. A 52-week study established the long-term safety of naldemedine [25]. Naldemedine was approved for OIC treatment based on the outcomes of this long-term study. Multiple studies show that naldemedine is effective and safe to use in cancer patients as well [26-27].

## Conclusions

OIC has transitioned from a poorly understood disorder to a well-defined clinical disorder. Major milestones have been achieved in defining OIC, elaborating its complex pathology and effect on the quality of life. Opioid therapy affects GI tract function, leading to changes in defecation patterns and bowel habits. Cancer patients taking opioids to treat OIC are likely to experience distress and poor quality of life. Existing and novel treatments for OIC have been studied comprehensively and validated. Stimulant and osmotic laxatives are the primary treatments for OIC. Novel treatments have been approved in the last few years, mainly for patients who fail to respond to conventional laxative treatment. PAMORAs are among the recently approved therapeutics and include methylnaltrexone, naloxegol, and naldemedine. Long-term safety and efficacy studies should be done carefully to ensure only effective therapeutics are approved. It is recommended to use the AGA OIC clinical decision tool when trying to approach a patient with OIC.
